# The Importance of Predictive Biomarkers and Their Correlation with the Response to Immunotherapy in Solid Tumors—Impact on Clinical Practice

**DOI:** 10.3390/biomedicines12092146

**Published:** 2024-09-22

**Authors:** Raluca Ioana Mihaila, Adelina Silvana Gheorghe, Daniela Luminita Zob, Dana Lucia Stanculeanu

**Affiliations:** 1 Department of Oncology, “Carol Davila” University of Medicine and Pharmacy, 020021 Bucharest, Romania; 2 Department of Medical Oncology I, “Prof. Dr. Alexandru Trestioreanu”, Institute of Oncology, 022328 Bucharest, Romania

**Keywords:** immunotherapy, biomarkers, TMB, MSI, ctDNA, local immunity, systemic immunity

## Abstract

**Background/Objectives**: Immunotherapy has changed the therapeutic approach for various solid tumors, especially lung tumors, malignant melanoma, renal and urogenital carcinomas, demonstrating significant antitumor activity, with tolerable safety profiles and durable responses. However, not all patients benefit from immunotherapy, underscoring the need for predictive biomarkers that can identify those most likely to respond to treatment. **Methods:** The integration of predictive biomarkers into clinical practice for immune checkpoint inhibitors (ICI) holds great promise for personalized cancer treatment. Programmed death ligand 1 (PD-L1) expression, tumor mutational burden (TMB), microsatellite instability (MSI), gene expression profiles and circulating tumor DNA (ctDNA) have shown potential in predicting ICI responses across various cancers. **Results**: Challenges such as standardization, validation, regulatory approval, and cost-effectiveness must be addressed to realize their full potential. Predictive biomarkers are crucial for optimizing the clinical use of ICIs in cancer therapy. **Conclusions**: While significant progress has been made, further research and collaboration among clinicians, researchers, and regulatory institutes are essential to overcome the challenges of clinical implementation. However, little is known about the relationship between local and systemic immune responses and the correlation with response to oncological therapies and patient survival.

## 1. Introduction

Carcinogenesis and tumor growth are associated with chronic inflammation and stimulation of the host’s immune system. The chronic systemic inflammatory response has been extensively investigated in relation to the progression and prognosis of specific tumors. Thus, immunomodulatory therapies have emerged as effective antineoplastic therapeutic strategies. Immunotherapy (check point inhibitors anti PD-1, PD-L1 or anti CTLA-4) has demonstrated significant antitumor activity, with tolerable safety profiles and durable responses in various neoplasia.

Currently, immunotherapy is used either in monotherapy/combination of immunotherapeutic agents or in addition to antineoplastic agents (chemotherapy, targeted therapy) in first-line treatment or subsequent lines, mainly in lung, melanoma, ENT, breast, bladder cancers, etc. The immune system is divided into two main branches: the innate immune system and adaptive immune system, with key elements serving a role in optimal functioning and have distinct, but complementary roles in ensuring optimal immune function [1]. Whether we are discussing the components of the innate immune system (physiological barriers, phagocytes, natural killer cells, dendritic cells or cytokines) or the adaptive one (helper T cells CD4+, cytotoxic T cells CD8+, regulatory T cells (Tregs), B lymphocytes, antibodies, memory lymphocytes), all have a well-defined role and contribute to antineoplastic defense [1,2]. While the innate immune system provides a rapid, non-specific response, the adaptive immune system provides a delayed but highly specific and memory-based response to pathogens [1,3].

The innate immune system is the first line of defense and provides a rapid, non-specific response to pathogens and does not require prior exposure to a pathogen to be effective [3]. The key elements of the innate immune system are physical and chemical barriers, specific immune cells, proteins, and molecules (complement system, cytokines, and chemokines) [1,3]. Physical and chemical barriers include the skin barrier, mucous membranes (respiratory, gastrointestinal, and urogenital tracts), secretions (saliva, tears, and mucus), acidic environment (low pH in the stomach or skin). Specific immune cells (macrophages, neutrophils, basophils, dendritic cells, and natural killer cells) have multiple roles to ensure the rapid innate immune response. Dendritic cells interact with antigens and present them to T cells in the adaptive immune system, serving as a bridge between innate and adaptive immunity [3,4]. Natural killer (NK) cells can identify and destroy infected or cancerous cells by recognizing changes in the expression of cell surface proteins [5]. Basophils release histamine and other chemicals during allergic reactions and inflammation, aiding in the recruitment of other immune cells [4]. The complement system, cytokines, and chemokines enhance the ability of antibodies and phagocytic cells to clear pathogens, promote inflammation, determine specific effects regarding interactions and communications between cells, and destroy the pathogen’s cell membrane [2,6].

The adaptive immune system provides a specific response to pathogens memorizing previous encounters, allowing for a faster and stronger response upon subsequent exposures [2]. It involves specialized cells that target specific antigens present on pathogens. Representative elements of the adaptive immune system include B lymphocytes, responsible for humoral immunity and T cells (helper T cells, cytotoxic T cells and regulatory T cells) [2]. B cells recognize specific antigens via their B cell receptors (BCRs) and can differentiate into plasma cells, which produce and secrete antibodies that neutralize pathogens or mark them for destruction, and memory B cells, providing long-term immunity by rapidly responding to future exposures to the same antigen [4,7]. 

T cells are responsible for cell-mediated immunity [2]. T cells recognize specific antigens via their T cell receptors (TCRs) and can differentiate into helper T cells (CD4+ T cells), cytotoxic T cells (CD8+ T cells) and cytotoxic T cells [2,4]. CD4+ T cells coordinate the immune response by secreting cytokines that activate other immune cells (B cells, macrophages, and cytotoxic T cells). CD8+ T cells directly induce the apoptosis of infected or cancerous cells by recognizing antigens presented by major histocompatibility complex (MHC) class I molecules. Regulatory T cells (Tregs) help maintain immune tolerance and prevent autoimmunity by suppressing excessive immune responses [2,4]. Both the innate and adaptive immune systems have distinct but complementary roles in ensuring optimal immune function. The innate and adaptive immune systems are integral to the body’s defense against pathogens. While the innate immune system provides rapid, non-specific responses, the adaptive immune system offers delayed but highly specific and memory-based responses. The interactions between these two systems ensure a comprehensive immune response that protects the host from infections and diseases while maintaining immune homeostasis [1,3,4]. 

Pathogens, including cancer cells, have evolved mechanisms to evade both innate and adaptive immune responses, such as antigenic variation, suppression of MHC expression, and resistance to complement activation [4,6,8]. Leveraging both arms of the immune system, especially through cancer immunotherapy (e.g., checkpoint inhibitors, CAR-T cell therapy) has been and remains a major focus of research [4]. By understanding the complex interplay between innate and adaptive immunity, researchers have developed novel therapies to enhance immune function, target diseases, and maintain overall health [4].

Malignant cells develop mechanisms to avoid the immune system, thus leading to uncontrolled proliferation, invasion and metastasis [9,10]. One of the most important mechanisms by which malignant cells achieve the inhibition of the immune system is the overexpression of the receptors of the immune control point—PDL1 (Programmed death ligand 1) or CTLA-4 [11,12]. Another important component in the interaction between neoplastic cells and the immune system is the tumor microenvironment (TME). Hanahan et al. described the hallmarks of cancer, emphasizing the dynamic interactions between cancer cells and their microenvironment [13]. TME is composed of different types of cells and extracellular components (extracellular matrix and stomal cells) that can suppress immune responses [14,15]. The TME often contains Tregs cells, myeloid-derived suppressor cells (MDSCs) and tumor-associated macrophages (TAMs) that produce immunosuppressive cytokines (IL-10, TGF-β) and inhibit T-lymphocyte function [16]. Intra-tumoral hypoxia can lead to the accumulation of adenosine, which suppresses the activity of T cells.

Intratumoral hypoxia is a common characteristic of many solid tumors, resulting from rapid cell proliferation, irregular vascularization, and abnormal metabolic processes [17]. Intratumoral hypoxia is defined as a condition of insufficient oxygen supply within the tumor mass, leading to an oxygen concentration of less than 2% and contributes to tumor progression, metastasis, and resistance to therapy by creating a hostile microenvironment that promotes tumor cell survival and impairs immune cell function [18]. One of the critical mechanisms through which hypoxia exerts immunosuppressive effects is through the accumulation of adenosine within TME. Adenosine is a potent immunosuppressive metabolite that can inhibit the activity of T cells, thereby allowing tumor cells to evade immune surveillance and destruction. Hypoxia can be caused by several factors, including rapid tumor growth, irregular tumor vasculature, and increased metabolic activity of tumor cells [19]. 

Under hypoxic conditions, tumor cells undergo several metabolic adaptations that result in the accumulation of adenosine [18,19,20]. Hypoxic tumor cells release large amounts of ATP into the extracellular space due to cellular stress and increased metabolic activity. The released ATP is rapidly converted into adenosine by the action of ecto-nucleotidases, such as CD39 (which converts ATP to ADP and AMP) and CD73 (which converts AMP to adenosine). Hypoxia upregulates hypoxia-inducible factors (HIF-1α and HIF-2α), which are transcription factors that enhance the expression of CD39 and CD73, promoting the accumulation of adenosine [21].

Adenosine is a potent immunosuppressive molecule that can inhibit various aspects of the T cell response, exerting its effects by binding to specific adenosine receptors (A1, A2A, A2B, and A3) expressed on the surface of the immune cells. Among these, the A2A receptor (A2AR) on T cells plays a critical role in mediating adenosine’s immunosuppressive effects [22,23,24,25,26]. Adenosine binding to A2AR on T cells triggers intracellular signaling pathways that increase cyclic AMP (cAMP) levels, leading to the inhibition of T cell receptor (TCR) signaling, proliferation, cytokine production (e.g., IFN-γ, IL-2), and cytotoxic activity. Prolonged exposure to adenosine in the TME can promote a state of T cell exhaustion characterized by the upregulation of inhibitory receptors (e.g., PD-1, CTLA-4) and a decrease in effector cytokine production. Adenosine promotes the differentiation and expansion of immunosuppressive regulatory T cells (Tregs), which further suppress the activity of effector T cells and other immune cells in the TME. Adenosine binding to A2AR on Tregs enhances their immunosuppressive capabilities, leading to increased secretion of anti-inflammatory cytokines such as IL-10 and TGF-β, which suppress effector T cell activity [27,28]. Adenosine inhibits the maturation and function of dendritic cells, which are crucial for antigen presentation and the activation of T cells. Adenosine-treated DCs show the reduced expression of costimulatory molecules (e.g., CD80, CD86) and decreased production of pro-inflammatory cytokines [29].

Given the role of adenosine in promoting an immunosuppressive TME, targeting adenosine signaling pathways has emerged as a potential therapeutic strategy in cancer immunotherapy [29]. Blocking adenosine receptors, particularly A2AR, with specific antagonists (e.g., CPI-444, AZD4635) can restore T cell function and enhance anti-tumor immunity. Targeting the enzymes responsible for adenosine production, such as CD39 and CD73, using monoclonal antibodies or small-molecule inhibitors can reduce adenosine levels in the TME and improve T cell activity [21,30]. Tumors may develop resistance to adenosine-targeted therapies through the upregulation of alternative immunosuppressive pathways or adaptive changes in the TME. Identifying reliable biomarkers for patient stratification and monitoring the response to adenosine-targeted therapies is critical for optimizing treatment [30].

Other mechanisms that generate immune escape include antigenic variation and loss, the downregulation of the MHC, immune modulation, enzymatic degradation, and resistance to apoptosis [31,32].

Antigenic variation and loss refer to the dynamic changes in the expression of antigens by tumor cells, allowing them to evade recognition and destruction by the immune system. This process is a major mechanism of immune evasion in cancer, contributing to tumor progression, metastasis, and resistance to immunotherapy. Tumor cells may undergo antigenic variation through genetic mutations, epigenetic modifications, or alterations in antigen processing and presentation pathways, leading to decreased visibility to immune cells. Tumor cells may acquire mutations in the genes encoding tumor-associated antigens (TAAs) or neoantigens. These mutations can alter the amino acid sequences of antigens, leading to the generation of variants that are less recognizable by the immune system [33]. High levels of chromosomal instability (CIN) in tumor cells can result in the deletion or amplification of genes encoding TAAs. This instability can cause the loss of antigen expression or the emergence of antigen-negative clones [34]. The immune system exerts selective pressure on tumors, favoring the survival of antigen-loss variants that are less recognizable by immune cells. This process, known as immunoediting, results in the outgrowth of tumor cells with reduced antigenicity. Mittal et al. (2014) described the three phases of cancer immunoediting—elimination, equilibrium, and escape—emphasizing the role of antigenic variation in immune escape [35].

The downregulation of the MHC, which is necessary for antigen presentation to T cells and immune modulation through the excretion of cytokines (TGF-β, IL-10 and VEGF) which inhibit the function and proliferation of T cells and other immune cells) are potent mechanisms that generate immune escape. Tumor cells often undergo epigenetic changes, such as DNA methylation and histone modifications, that silence the expression of genes encoding TAAs or molecules involved in antigen processing and presentation (e.g., MHC class I molecules) [36]. Other immune evasion patterns suggest inadequate enzymatic degradation (indoleamine 2,3-dioxygenase/IDO can degrade tryptophan, useful for T cell function, leading to immune suppression) and resistance to apoptosis (overexpression of BCL-2 and BCL-Xl proteins and Fas receptor mutation). Enzymes such as matrix metalloproteinases (MMPs), indoleamine 2,3-dioxygenase (IDO), and other proteolytic enzymes play vital roles in the regulation of immune activity. Tumors often manipulate these enzymes to maintain an immunosuppressive environment, promote immune tolerance, and enhance tumor progression [37]. Resistance to apoptosis is a key hallmark that allows cancer cells to survive despite accumulating genetic abnormalities, oncogenic stress, and exposure to cytotoxic therapies [13].

It could be useful to determine the degree of infiltration of lymphocytes in the tumor microenvironment in relation to local or systemic immunity, hence the prognostic value in different neoplasia [38]. Tumor lymphocytic infiltrates (TILs) have been studied as an indicator of tumor inflammation and TIL subsets have been reported to have their own roles in cancer progression in colorectal and gastric cancer [39,40,41]. Data available emphasizes that CD8 + TILs or an increased TIL infiltrate are associated with a favorable clinical prognosis in lung cancer [38], melanoma [42] and breast cancer [43,44]. Erdag et al. found a strong correlation between high levels of TILs and improved survival in metastatic melanoma [45]. The presence and density of TILs have significant prognostic implications in breast cancer, particularly in specific subtypes like triple-negative breast cancer (TNBC) and HER2+ breast cancer, due to their higher immunogenicity compared to hormone receptor-positive (HR+) breast cancer. High levels of TILs are associated with improved overall survival (OS) and disease-free survival (DFS) in patients with TNBC. This subtype often has higher mutational loads, leading to increased neoantigen formation, which can enhance TIL recruitment. García-Teijido P et al. reported that a 10% increase in TILs correlates with a 15–20% reduction in the risk of recurrence or death in early-stage TNBC [46]. Similar to TNBC, high levels of TILs in HER2+ breast cancer are linked to better survival outcomes and higher response rates to HER2-targeted therapies (e.g., trastuzumab). Xiu T. et al. demonstrated that TILs are predictive of a favorable response to trastuzumab in HER2-positive breast cancer [47].

Peripheral blood parameters (Table 1) emerged as indicators of the host’s immune response, and preclinical and clinical data have suggested that circulating white blood cells, resulting in a change in the proportions of neutrophils, lymphocytes and monocytes, may be associated with systemic inflammatory responses [48,49]. Their dosage is easy to use and less expensive in measuring systemic immunity. Leukocytosis is frequently found in neoplastic diseases. The hypothesis that leukocytosis is a negative prognostic factor has been stated in several prospective and retrospective analyses [50,51,52]. Leukocytes are a heterogeneous population of blood cells. Therefore, the subpopulation of leukocytes responsible for the unfavorable prognostic impact should be identified. Roh et al. emphasized that leukocytosis is associated with advanced tumor disease and increased recurrence and mortality patients with oral cavity squamous cell carcinoma [51]. The relationships between neoplasia prognosis and total monocyte count (AMC), absolute lymphocyte count (ALC), lymphocyte–monocyte ratio (LMR), neutrophil–lymphocyte ratio (NLR), and platelet–lymphocyte ratio (PLR) have been studied in lung cancer, sarcomas, breast, thyroid, gastric, colorectal, and oral cavity squamous cell carcinoma [53,54,55,56,57,58,59,60,61,62]. Diem et al. stated the fact that elevated pre-treatment NLR and PLR are associated with shorter OS and progression-free survival (PFS) and with lower response rates in patients with metastatic non-small cell lung cancer treated with nivolumab independently of other prognostic factors [57]. It has been hypothesized that neutrophilia and lymphocytopenia are negative factors for patients diagnosed with lung and ENT neoplasms [59,62]. Other inflammatory markers, such as VSH, fibrinogen, CRP, various cytokines and other proteins involved in the inflammatory process and immunity, can provide important data related to the tumor microenvironment and the immunological characteristics of the patient [31].

By approaching the therapeutic targets identified in the deficient pattern of the immune system, in the presence of neoplastic cells through innovative therapies, researchers aimed to improve the effectiveness of immunotherapies and obtain lasting responses with a maintained quality of life [63]. Immune checkpoint inhibitors have the role of restoring the activity of T cells against malignant cells, blocking the PD-1/PD-L1 interaction or restoring the antitumor activity of T cells (anti-CTLA4 therapies) [9,12]. Both the immune response and the reduction in the resistance to antineoplastic therapies can be obtained by combining biological therapies (immunotherapy or targeted therapy) and chemotherapy or radiotherapy. Among the new immune therapies is CAR T cell therapy, genetically modified cells that express chimeric antigen receptors (CAR) and are designed to target specific tumor antigens, bypassing some immune evasion mechanisms. Another antineoplastic mechanism was identified to be the modulation of the tumor microenvironment, through antiangiogenic and extracellular matrix remodeling therapies, vaccines and oncolytic viruses and adoptive cell transfer [12].

## 2. Predictive Biomarkers for Immunotherapy and Their Prognostic Role Regarding Treatment Response

Immunotherapy has revolutionized the treatment landscape for various solid tumors, offering new hope for patients who previously had limited options. However, not all patients respond to immunotherapy, underscoring the need for predictive biomarkers that can identify those most likely to benefit. Serum biomarkers are particularly attractive due to their minimally invasive nature and ease of repeated measurement. Among most investigated serum biomarkers are PD-L1 [12], tumor mutation burden [63], microsatellite instability [64], elements from peripheral blood leukocytes, neutrophils, eosinophils, basophils, platelets, monocyte count (AMC), absolute lymphocyte count (ALC), lymphocyte–monocyte ratio (LMR), neutrophil–lymphocyte ratio (NLR) and platelet–lymphocyte ratio (PLR) [53,54,55,56,57,58,59,60,61,62], and other inflammatory markers such as VSH, fibrinogen, CRP (C-reactive protein), and various cytokines [12], as per Table 2.

## 3. PD-L1 Expression

Programmed death-ligand 1 (PD-L1) expression on tumor cells has been one of the most extensively studied biomarkers for predicting the response to immune checkpoint inhibitors (ICIs) [67]. The blockade of the PD-1/PD-L1 pathway with immune checkpoint inhibitors (ICIs) has revolutionized cancer therapy, especially in malignancies like melanoma, non-small cell lung cancer (NSCLC), and bladder cancer [67,68,69,70]. High-PD-L1 expression correlates with better responses to immunotherapy in various cancers, including non-small cell lung cancer (NSCLC) and melanoma. Kluger et al. published data demonstrating the fact that PD-L1 expression in melanoma tumor cells is lower than NSCLC or RCC cells [67]. The higher response rate in melanoma patients treated with PD-1 inhibitors is likely related to PD-L1 in tumor-associated inflammatory cells [71]. However, PD-L1 testing is not universally predictive and may not fully capture the complex tumor-immune microenvironment [64,72]. PD-L1, expressed on tumor cells and tumor-infiltrating immune cells, binds to the PD-1 receptor on T cells, leading to the inhibition of T cell activity [67,68]. This mechanism allows tumors to evade the immune response. Blocking this interaction with ICIs can restore T cell activity and enhance anti-tumor immunity. In non-small cell lung cancer (NSCLC), PD-L1 expression levels are used to stratify patients for treatment with pembrolizumab, where those with higher expression (≥50%) have shown better outcomes compared to chemotherapy [64,72,73]. PD-L1 expression can be heterogeneous within tumors and may change over time or in response to treatments, posing challenges for its use as a sole predictive biomarker [72]. Although PD-L1 is a critical biomarker, it is not the only one. Tumor mutational burden (TMB), microsatellite instability (MSI), and gene expression profiles are also being studied to enhance the predictive accuracy of ICIs [65,74,75]. Combining PD-L1 expression with these biomarkers can provide a more comprehensive prediction of treatment response. This indicates the need for further research to identify additional biomarkers and better understand the tumor-immune microenvironment. The FDA has approved several ICIs along with companion diagnostics to measure PD-L1 expression, like the PD-L1 IHC 28-8 pharmDx assay and the VENTANA PD-L1 (SP142) assay. The integration of PD-L1 expression with other biomarkers and advanced diagnostic techniques, such as multiplex immunohistochemistry and next-generation sequencing, is expected to improve the selection of patients for ICIs [72]. Continued research into the mechanisms of immune evasion and resistance will likely yield new biomarkers and therapeutic targets, enhancing the efficacy and personalization of cancer immunotherapy.

## 4. Tumor Mutational Burden (TMB)

TMB refers to the number of mutations within a tumor’s genome. High TMB has been associated with increased neoantigen load, which can enhance the immune system’s ability to recognize and attack tumor cells [65]. High-TMB tumors may respond better to ICIs across different cancer types [74,75]. However, the standardization of TMB measurement and its application in clinical practice remain challenging. Data available from patients with melanoma, non-small cell lung cancer (NSCLC), and urothelial carcinoma have demonstrated that patients with high TMB show improved outcomes when treated with ICIs such as pembrolizumab and nivolumab [76,77]. The rationale behind TMB as a predictive biomarker lies in the generation of neoantigens from tumor mutations. Higher mutation rates increase the likelihood of neoantigen formation, which can be recognized by the immune system, thus enhancing the efficacy of ICIs. Neoantigens are essential for T cell activation and tumor infiltration, making TMB a delegate for the immunogenicity of the tumor [33].

While TMB has shown promise in predicting responses, its predictive value is not universal. In breast cancer and prostate cancer, the correlation between TMB and ICI response is less clear, potentially due to the lower overall mutation rates in these cancers. Goodman et al. highlighted that TMB is a robust predictor in cancers with high mutation rates, such as melanoma and NSCLC, but not as effective in cancers with low mutation rates [65]. The methods for measuring TMB vary across studies, with whole-exome sequencing (WES) and targeted gene panels being the most common. However, the lack of standardization in TMB quantification poses challenges for its clinical application. Efforts are underway to standardize TMB measurement and establish cutoff values that can reliably predict ICI responses [78]. Combining TMB with other biomarkers such as PD-L1 expression [72] may improve the predictive accuracy for ICI responses. A multifactorial approach incorporating TMB and PD-L1 expression provides a more comprehensive assessment of tumor immunogenicity and potential response to ICIs. However, the variability in TMB’s predictive value across different cancers and the lack of standardized measurement methods highlight the need for further research [79]. TMB holds significant promise as a biomarker for predicting responses to ICIs, particularly in cancers with high mutation rates. Future studies should focus on standardizing TMB measurement and exploring its combined use with other biomarkers to improve the prediction of ICI efficacy.

## 5. Microsatellite Instability (MSI)

MSI refers to the condition of genetic hypermutability that results from impaired DNA mismatch repair (MMR). This condition leads to the accumulation of mutations, particularly in microsatellite regions. MSI is a hallmark of certain cancers, most notably colorectal cancer (CRC), but it is also found in endometrial, gastric, and other cancer types [80,81]. Tumors with high levels of MSI (MSI-High or MSI-H) are characterized by a high mutation burden and are more likely to produce neoantigens that make them recognizable to the immune system (increased immunogenicity), rendering them potentially more responsive to ICIs. Thus, MSI has emerged as a significant biomarker for predicting the response to ICIs [82,83,84]. MSI-high status is associated with a positive response to ICIs, particularly in colorectal cancer [85,86]. Casak SJ emphasized the data from the Keynote-177 study, which showed that pembrolizumab induced durable responses in patients with MSI-H colorectal cancer, with an impact on OS and PFS (Study Keynote-177) [66]. Pembrolizumab led to significantly longer progression-free survival than chemotherapy when received as first-line therapy for MSI-H–dMMR metastatic colorectal cancer, with fewer treatment-related adverse events. This led to the FDA approval of pembrolizumab for MSI-H or MMR-deficient (dMMR) metastatic colorectal cancer [87].

The efficacy of ICIs in MSI-H tumors is believed to be due to the high mutational load and the resulting increased production of neoantigens, which enhance the immune system’s ability to recognize and induce cancer cell apoptosis [81,82,83,88]. MSI-H tumors often exhibit an inflammatory microenvironment with high levels of immune cell infiltration, which may also contribute to the enhanced response to ICIs. Overman et al. reported that nivolumab demonstrated significant antitumor activity in MSI-H metastatic colorectal cancer [89].

MSI status can be determined using various methods, including PCR-based assays and next-generation sequencing (NGS). Immunohistochemistry (IHC) for MMR proteins is also used as a surrogate marker for MSI. Standardizing these diagnostic methods is crucial for accurately identifying MSI-H patients who may benefit from ICIs. Combining MSI with other biomarkers like TMB and PD-L1 expression can enhance the predictive power for ICI responses [65,74,75]. The identification of MSI-H status has significant clinical implications, as it can guide treatment decisions and improve patient outcomes. The current data support the role of MSI-H status as a predictive biomarker for ICI efficacy across various cancer types, especially CRC [90]. However, the variability in response among different cancer types and the lack of standardization in diagnostic methods present challenges that need to be addressed [81,82,83,84,88].

## 6. Novel Serum Biomarkers

Cytokines and chemokines play crucial roles in the immune response and tumor microenvironment. Elevated levels of certain cytokines, such as interleukin-6 (IL-6) and C-reactive protein (CRP), have been linked to poorer outcomes in cancer patients receiving immunotherapy [91,92,93].

IL-6 promotes cancer cell proliferation, survival, and metastasis through the activation of the JAK/STAT3, PI3K/AKT, and MAPK signaling pathways. It also supports a suppressive TME by expanding regulatory T cells (Tregs) and myeloid-derived suppressor cells (MDSCs), both of which inhibit effective anti-tumor immunity [94]. Elevated IL-6 levels have been linked to resistance to ICIs such as anti-PD-1/PD-L1 and anti-CTLA-4 antibodies. Patients with high IL-6 levels often exhibit lower response rates and poorer overall survival [91,92,93,94,95].

CRP is an acute-phase protein produced by the liver in response to inflammation and is often elevated in cancer patients with systemic inflammation. Elevated CRP levels have been associated with poor prognosis and decreased survival in patients undergoing immunotherapy [93,96]. CRP is often considered a surrogate marker for IL-6 activity since its production is primarily induced by IL-6. High CRP levels reflect systemic inflammation and are linked to a pro-tumorigenic TME, characterized by immunosuppressive cells, such as Tregs and MDSCs. Elevated CRP levels can indicate a less favorable response to ICIs. CRP may be used alongside other markers, like IL-6, to better stratify patients who are less likely to benefit from immunotherapy [93,95].

L-8 is a pro-inflammatory chemokine that promotes angiogenesis, tumor growth, and metastasis by recruiting neutrophils and MDSCs to the TME. Elevated IL-8 levels have been associated with poor outcomes in patients receiving ICIs [96,97]. TGF-β is a cytokine with a dual role in cancer. While it can inhibit early tumor development, in established tumors, it promotes immune evasion, metastasis, and fibrosis. High levels of TGF-β are associated with resistance to ICIs [98].

Circulating tumor DNA (ctDNA) offers a non-invasive means to assess tumor genetics and monitor disease progression. Changes in ctDNA levels during treatment can provide insights into the effectiveness of immunotherapy and early indications of resistance [96,99]. As a non-invasive test, ctDNA can provide real-time insights into the molecular profile of a tumor, including the presence of specific mutations, TMB, and other genomic alterations. These characteristics make ctDNA a promising biomarker for predicting responses to cancer immunotherapy, particularly with ICIs targeting PD-1, PD-L1, and CTLA-4 [99]. Lower baseline levels of ctDNA have been associated with better responses to ICIs in multiple cancer types. Patients with low ctDNA levels often have lower tumor burden and less aggressive disease, which correlates with better outcomes following immunotherapy [100]. Complete clearance of ctDNA during immunotherapy has been correlated with complete tumor regression and long-term survival. Conversely, persistent or rising ctDNA levels may indicate minimal residual disease (MRD) or early progression, suggesting that ctDNA can serve as a surrogate marker for treatment response and prognosis [101]. ctDNA can detect response or progression earlier than radiographic imaging. This early detection capability can help clinicians make timely decisions regarding the continuation, modification, or discontinuation of therapy [102]. There is currently a lack of standardized protocols for ctDNA testing, including differences in sample processing, DNA extraction methods, and sequencing platforms. Variability in these factors can affect the sensitivity and specificity of ctDNA as a predictive biomarker [103].

## 7. Discussions

The integration of serum biomarkers into clinical practice involves several steps: standardization, validation and accessibility [104]. Uniform methodologies for biomarker assessment are needed to ensure consistency and reliability across different laboratories. Prospective clinical trials are essential to validate the predictive value of emerging biomarkers. Also, cost-effective and accessible testing methods are crucial for widespread implementation in clinical settings [13]. Predictive biomarkers are essential for identifying the patients who are most likely to benefit from ICIs, thereby optimizing treatment outcomes and minimizing unnecessary side effects [11]. PD-L1 expression on tumor cells is one of the most widely studied predictive biomarkers for ICIs. High PD-L1 expression is generally associated with better responses to anti-PD-1 and anti-PD-L1 therapies in melanoma or NSCLC. Atezolizumab, pembrolizumab, and nivolumab have shown improved efficacy in patients with high PD-L1 expression in various clinical trials [105,106,107]. However, PD-L1 testing lacks standardization, with different assays and cut-off values, making clinical implementation challenging [72]. TMB measures the number of mutations per mega base of tumor DNA, serving as an indicator of neoantigen load. High TMB is associated with better responses to ICIs across multiple cancer types, including melanoma, NSCLC, and bladder cancer. Clinical trials, such as the Checkmate 848 study, have demonstrated the predictive value of TMB for ICI response [108]. Implementing TMB testing in clinical practice requires standardized methodologies and validated thresholds [77]. MSI is a condition of genetic hypermutability that results from impaired DNA mismatch repair. MSI-H is predictive of ICI efficacy, particularly in colorectal cancer [66]. The FDA has approved pembrolizumab for MSI-H or mismatch-repair-deficient (dMMR) tumors, regardless of tumor type, highlighting its clinical utility [109]. MSI testing is well established and integrated into clinical workflows for certain cancers. Gene expression profiling can identify immune-related signatures predictive of ICI response. ctDNA provides a non-invasive method to monitor tumor dynamics and predict ICI responses. Dynamic changes in ctDNA levels during treatment can indicate response or resistance to ICIs. Studies have shown that decreasing ctDNA levels are associated with better outcomes, while stable or increasing levels suggest poor responses [101,103]. Standardizing ctDNA testing and integrating it into clinical practice remain challenges. There are also challenges in clinical implementation in current practice: lack of standardization in biomarker testing methodologies and cut-offs complicates clinical implementation, demonstrating that the clinical utility and cost-effectiveness of predictive biomarkers is crucial for their adoption in routine practice, regulatory approval and reimbursement policies vary across regions, affecting biomarker adoption, combining multiple biomarkers (e.g., PD-L1, TMB, and MSI) may enhance predictive accuracy, but requires complex testing algorithms and interpretation.

## 8. Conclusions and Perspective Directions

It seems that a single biomarker is not enough to identify patients responsive to immunotherapy. Therefore, a correlation between them could be useful. For the future, liquid biopsy, which is a non-invasive diagnostic procedure and involves the isolation of circulating biomarkers such as circulating tumor cells, cell-free DNA (cfDNA) and microRNA (miRNA), would be very useful considering recent advances (such as genomic sequencing (NGS) and PCR-type analysis) for providing basic information about tumors and monitoring the response to therapies. However, everything involves very high costs and is not applicable in real life. To ensure that the measurement of the usual inflammatory biomarkers is simple and convenient in the long term, the purpose of the present review was to discuss the role of each biomarker and their ratio and validation for clinical practice in evaluating the response to checkpoint inhibitors. Serum biomarkers hold significant promise for predicting responses to immunotherapy in solid tumors, potentially guiding treatment decisions and improving patient outcomes. While PD-L1, TMB, and MSI are currently the most established biomarkers, ongoing research into biomarkers from peripheral blood cytokines, ctDNA, and immune cell profiles may expand the repertoire of useful biomarkers. As our understanding of the tumor-immune interplay deepens, the clinical application of these biomarkers will likely become an integral part of personalized cancer therapy.

The integration of predictive biomarkers into clinical practice for ICI therapy holds great promise for personalized cancer treatment. PD-L1 expression, TMB, MSI, gene expression profiles, and ctDNA have shown potential in predicting ICI responses across various cancers. However, challenges such as standardization, validation, regulatory approval, and cost-effectiveness must be addressed to realize their full potential.

Predictive biomarkers are crucial for optimizing the clinical use of ICIs in cancer therapy. While significant progress has been made, further research and collaboration among clinicians, researchers, and regulatory institutes are essential to overcome the challenges of clinical implementation. Standardizing biomarker testing, validating predictive algorithms, and ensuring cost-effective integration into clinical workflows will be key to enhancing personalized cancer treatment and improving patient outcomes.

The characterization of the patient’s immune parameters, but also of the tumor microenvironment before the initiation of treatment, can identify predictive biomarkers for clinical and paraclinical response and can define the subgroup of patients who do not respond to immunotherapy. It is also necessary to identify prognostic markers for patients who are indicated for immunotherapy, and which can be useful for monitoring patient responses or IRAEs. However, clinical predictive factors for treatment responses or IRAE risk remain unclear. Establishing the predictive relationship between the immune characteristics of patients, the tumor microenvironment and paraclinical biomarkers, and the response to immunotherapy treatments, as well as identifying prognostic biomarkers regarding survival, quality of life, and the risk of adverse immune events, should be addressed in future studies to identify the right treatment for the right patient.

## Figures and Tables

**Table 1:** Hematologic peripheral blood biomarkers.

Biomarker	Prognostic
Leukocytosis	Unfavorable
Neutrophilia	Unfavorable
Lymphocytopenia	Unfavorable
Eosinophils	Favorable
Thrombocytosis	Unfavorable
Basophilia	Unclear

**Table 2:** Summarization of the predictive role of the most frequent biomarkers.

Biomarker	Value	Predictive Role	Reference
PD-L1	High	Better responses to immnutherapy in various cancers, including non-small cell lung cancer (NSCLC) and melanoma	Havel, J. J., Chowell, D., and Chan, T. A. (2019) [12]
Tumor mutation burden (TMB)	High	Favorable responses to ICIs across different cancer types	Goodman, A. M., et al. (2017) [65]
Microsatellite instability-MSI	High	MSI-H status has been associated with better responses to ICIs	Casak SJ. (2021) [66]
Neutrophil–lymphocyte ratio (NLR)	High	Poor prognosis and lower response rates to ICIs	Diem et al. (2017) [57]
